# When Metabolic Dysfunction-Associated Steatotic Liver Disease Meets Viral Hepatitis

**DOI:** 10.3390/jcm14103422

**Published:** 2025-05-14

**Authors:** Imran Hasanoglu, Antonio Rivero-Juárez, Gülşen Özkaya Şahin, ESCMID Study Group for Viral Hepatitis (ESGVH)

**Affiliations:** 1 Department of Infectious Disease and Clinical Microbiology, Ankara Yildirim Beyazit University, Ankara Bilkent City Hospital, Ankara 06800, Türkiye; 2 Department of Infectious Diseases, Hospital Universitario Reina Sofia, Instituto Maimonides de Investigación Biomédica de Córdoba (IMIBIC), Universidad de Córdoba (UCO), 14071 Cordoba, Spain; arjvet@gmail.com; 3 Centro de Investigación Biomédica en Red (CIBER) Área de Enfermedades Infecciosas (CIBERINFEC), Instituto de Salud Carlos III (ISCIII), 28029 Madrid, Spain; 4 Clinical Microbiology, Infection Prevention and Control, Office for Medical Services, 22467 Lund, Sweden; gulsen.ozkaya_sahin@med.lu.se; 5 Division of Medical Microbiology, Department of Laboratory Medicine Lund, Medical Faculty, Lund University, 22467 Lund, Sweden

**Keywords:** viral hepatitis, MASLD, HBV, HCV, steatosis

## Abstract

The interplay between Metabolic Dysfunction-Associated Steatotic Liver Disease (MASLD) and viral hepatitis, primarily hepatitis B virus (HBV) and hepatitis C virus (HCV), presents a complex challenge in managing chronic liver diseases. Recent epidemiological insights suggest an escalating prevalence of MASLD globally, attributed mainly to the obesity epidemic and associated metabolic disorders. Concurrently, chronic viral hepatitis remains a significant contributor to liver disease morbidity and mortality worldwide, despite advances in antiviral therapies. According to the World Health Organization (WHO) 2023 data, approximately 296 million people are living with chronic HBV infection (about 3.8% of the global population), and 58 million people with HCV infection (about 0.7%), together accounting for over 1.1 million deaths annually. The coexistence of MASLD and viral hepatitis presents a complex scenario in clinical outcomes, where the effects on liver health can vary. Although many studies highlight the potential for additive or synergistic worsening of liver conditions, leading to complications such as cirrhosis, liver failure, and HCC, the impact of HBV on MASLD is not consistent. Managing patients with dual MASLD and viral hepatitis is complex due to the interplay of metabolic and viral factors. Lifestyle modifications, including weight loss, dietary changes, and physical activity, are fundamental to MASLD management and help reduce fibrosis risk in viral hepatitis. This review examines the dual impact of MASLD and viral hepatitis on liver pathology and delineates shared pathophysiological mechanisms, including the influence on hepatic steatosis, inflammation, and fibrogenesis. It also discusses therapeutic strategies tailored to manage this comorbidity, emphasizing the need for an integrated care approach that addresses both metabolic dysfunctions and viral infection to optimize patient outcomes.

## 1. Introduction

Metabolic Dysfunction-Associated Steatotic Liver Disease (MASLD) has emerged as one of the most pressing global health challenges of the 21st century. The rising prevalence of obesity, type 2 diabetes, and metabolic syndrome has made MASLD the most common chronic liver disease globally, affecting around 25% of the population [1]. Without proper management, MASLD can advance to more severe stages, such as steatohepatitis, cirrhosis, and hepatocellular carcinoma (HCC) [2,3]. Chronic viral hepatitis, caused primarily by hepatitis B virus (HBV) and hepatitis C virus (HCV), continues to be a major contributor to global liver disease morbidity and mortality. Despite significant advances in antiviral therapies, particularly for HCV, viral hepatitis remains a leading cause of liver-related complications, including fibrosis, cirrhosis, and HCC. In regions with high HBV endemicity, such as East Asia and sub-Saharan Africa, chronic HBV infection affects nearly 10% of the population, while HCV remains prevalent in many parts of the world, including high-income countries with a history of injection drug use or unsafe medical practices [4,5].

The interaction between HBV and MASLD is complex, with studies showing both worsening and protective effects on liver steatosis [6]. In contrast, HCV consistently promotes liver steatosis [7]. This contrasting clarity in the influence of HCV compared to the ambiguous effects of HBV illuminates the diverse mechanisms through which viral infections can interact with metabolic dysfunctions in the liver. Recognizing these differences is key to designing personalized therapeutic strategies that better address the needs of patients with viral hepatitis and metabolic complications. A tailored approach can enhance disease management and improve clinical outcomes. This review examines the intersection of MASLD and viral hepatitis, focusing on epidemiological trends, shared pathophysiological pathways, clinical outcomes, and management strategies. By reviewing current evidence, we aim to highlight the challenges of this dual condition and explore opportunities for improved patient care.

## 2. Epidemiological Insights

MASLD is now a leading cause of chronic liver disease, affecting approximately 25% of the global population [7]. Among HBV-infected patients, studies report MASLD prevalence rates ranging from 30 to 40% [8]. In HCV, the overlap is even more pronounced, reflecting the direct steatogenic effects of the virus [3]. HCV infection induces hepatic steatosis, with prevalence estimates ranging from 40% to 80%. When factors like obesity, diabetes, and alcohol use are excluded, the prevalence drops to around 40%, highlighting the role of both viral and host metabolic factors in liver steatosis [6].

Geographic disparities in the co-occurrence of MASLD and viral hepatitis underscore the influence of regional health trends. In East Asia, endemic HBV combined with rising obesity rates is driving an increase in dual-pathology cases [9]. Meanwhile, in Western countries, metabolic factors are the primary drivers of liver disease, complicating HCV eradication efforts amid rising MASLD prevalence [10]. Demographic factors such as age, sex, and comorbidities significantly influence the interplay between MASLD and viral hepatitis. Older age and male sex are consistently associated with higher fibrosis risks in co-affected individuals. Additionally, the presence of conditions required to define MASLD, such as type 2 diabetes, hypertension, and dyslipidemia, underscores the enhanced progression of liver disease in viral hepatitis patients [11]. Genetic factors also play a crucial role; the geographic variation in genes like PNPLA3 and TM6SF2 polymorphisms underscores individual susceptibility to this dual burden [12].

The global health implications of MASLD and viral hepatitis extend beyond their direct effects on liver health. For instance, the rising incidence of HCC in regions undergoing epidemiological transitions reflects the combined influence of viral and metabolic risk factors [13]. Population-based studies also reveal increased healthcare utilization and economic costs associated with dual-etiology liver disease [14]. These findings highlight the need for integrated strategies targeting both metabolic and viral contributors to liver disease.

## 3. Pathophysiology and Mechanisms

The pathophysiological interplay between MASLD and viral hepatitis is complex and multifactorial, reflecting both shared and distinct contributions to liver injury. This interaction is characterized by a convergence of metabolic and viral factors.

Both HCV and HBV interfere with lipid metabolism, but through distinct mechanisms. HCV directly contributes to hepatic steatosis by promoting lipid droplet formation and disrupting normal lipid metabolism. This occurs via the upregulation of lipogenic enzymes and reduced fat breakdown. HCV proteins enhance triglyceride synthesis and accumulation within hepatocytes, leading to steatosis, particularly in genotype 3 infections, which downregulate microsomal triglyceride transfer protein (MTP), impairing VLDL secretion [15,16]. On the other hand, HBV disrupts lipid homeostasis primarily through interactions between viral proteins and host lipid-related pathways. HBV X protein (HBx), in particular, has been implicated in modifying the expression of genes involved in lipid metabolism and transport, contributing to altered lipid profiles in infected hepatocytes [17,18].

MASLD amplifies metabolic disturbances in the liver by fostering a pro-inflammatory and pro-fibrotic environment, leading to a vicious cycle of lipid accumulation and hepatocyte injury. The presence of metabolic factors like insulin resistance, obesity, and type 2 diabetes enhances this cycle by increasing the influx of free fatty acids to the liver, further promoting lipid accumulation and sensitizing hepatocytes to viral and immune-mediated damage [19]. The metabolic environment also modulates viral replication dynamics. HCV proteins interfere with insulin signaling pathways, leading to insulin resistance, which in turn promotes hepatic de novo lipogenesis, further exacerbating steatosis. For instance, insulin resistance has been associated with increased HCV replication, while changes in lipid profiles may affect HBV replication [20,21,22].

These mechanisms highlight the complex interplay between viral and metabolic factors in MASLD. The complexity of these interactions underscores the need for integrated approaches in the therapeutic management of affected individuals, incorporating both antiviral strategies and interventions aimed at correcting metabolic imbalances. Recent studies suggest that epigenetic modifications, such as DNA methylation and histone alterations, play a key role in mediating this interaction. These findings highlight the intricate interplay between viral and metabolic factors, underscoring the need for integrated therapeutic approaches [23,24].

## 4. Clinical Outcomes

The coexistence of MASLD and viral hepatitis presents a complex scenario in clinical outcomes, where the effects on liver health can vary. Although many studies highlight the potential for additive or synergistic worsening of liver conditions, leading to complications such as cirrhosis, liver failure, and HCC, the impact of HBV on MASLD is not consistent [22,25,26]. The progression of fibrosis is accelerated in patients with MASLD and viral hepatitis, driven by the convergence of metabolic and viral fibrogenic pathways. In chronic hepatitis B, viral proteins such as HBx have been shown to upregulate fibrogenic mediators, including transforming growth factor-beta (TGF-β), connective tissue growth factor (CTGF), and platelet-derived growth factor (PDGF), thereby promoting hepatic stellate cell (HSC) activation and extracellular matrix deposition. Similarly, in chronic hepatitis C, persistent viral replication and immune-mediated injury lead to oxidative stress and the release of pro-inflammatory cytokines such as tumor necrosis factor-alpha (TNF-α), interleukin-1β (IL-1β), and interleukin-6 (IL-6), which further drive HSC activation and fibrogenesis. These viral fibrotic mechanisms, when combined with the pro-fibrogenic effects of metabolic dysfunction—including insulin resistance, lipotoxicity, and chronic low-grade inflammation—result in accelerated liver fibrosis and increased risk of cirrhosis in co-affected patients [27,28,29]. Histological studies reveal distinctive patterns of fibrosis in co-affected patients, including more extensive pericellular and septal fibrosis. Advanced fibrosis and cirrhosis are associated with higher rates of decompensation, portal hypertension, and liver transplantation in this population [30]. These findings underscore the importance of early fibrosis assessment using non-invasive tools such as transient elastography and serum biomarkers.

While limited direct comparative data exist, emerging evidence suggests that the temporal sequence of MASLD and viral hepatitis onset may significantly influence disease progression and clinical outcomes. In patients who develop MASLD following chronic HCV infection—particularly with genotype 3—viral-induced steatosis is a well-established phenomenon that contributes to faster fibrosis progression and a heightened risk of HCC due to the synergistic effects of metabolic and viral injury [3,15,31]. Additionally, MASLD may re-emerge or worsen after successful antiviral treatment with direct-acting antivirals (DAAs), likely driven by post-SVR weight gain, deterioration in insulin sensitivity, and unaddressed metabolic comorbidities [6]. A recent population-based study further supports this by showing that metabolic risk factors such as obesity, type 2 diabetes, and hypertension are independently associated with accelerated fibrosis in HCV-infected individuals with MASLD [32]. Alternatively, in patients with pre-existing MASLD who subsequently acquire HBV or HCV infection, disease progression may also be accelerated. In this context, metabolic dysfunction—including insulin resistance and lipotoxicity—creates a pro-inflammatory hepatic milieu that amplifies virus-induced hepatocellular injury and fibrogenesis [20,21]. Insulin resistance in particular has been shown to promote hepatic de novo lipogenesis and facilitate HCV replication, while also diminishing treatment response rates, especially in interferon-based regimens [21]. Although data on HBV are more heterogeneous, some reports suggest that MASLD may contribute to hepatocarcinogenesis even in the absence of advanced fibrosis, indicating a possible fibrosis-independent oncogenic synergy [33]. Meanwhile, other studies have not found a significant association between MASLD and fibrosis progression in chronic HBV, suggesting that viral–metabolic interactions may differ by etiology [34]. These bidirectional and etiology-specific interactions emphasize the need to account for disease temporality when evaluating liver disease severity and tailoring management in patients with dual etiologies.

HCC risk is markedly elevated in patients with MASLD and viral hepatitis, particularly those with advanced fibrosis or diabetes [31,35]. Studies indicate that MASLD contributes to HCC through metabolic inflammation, oxidative stress, and genomic instability, while viral hepatitis adds a layer of oncogenic potential via direct viral effects and chronic immune activation [28,29,36]. The combination of these factors creates a tumorigenic microenvironment, resulting in increase in HCC incidence compared to mono-etiology cases [31]. Additionally, steatosis-associated changes in the tumor microenvironment may influence the efficacy of HCC treatments, highlighting the need for tailored therapeutic approaches. Patients with MASLD and viral hepatitis face increased liver-related and all-cause mortality [31]. Cardiovascular disease, a common comorbidity in MASLD, further contributes to this heightened mortality risk, reflecting the systemic nature of metabolic dysfunction. Longitudinal studies highlight the need for comprehensive management strategies that address both hepatic and extrahepatic complications in these patients.

In addition to increased mortality, the dual burden of MASLD and viral hepatitis imposes significant psychosocial and economic challenges. Patients frequently report diminished quality of life due to chronic symptoms like fatigue and abdominal discomfort, compounded by the stress of managing multiple chronic conditions. The economic burden of frequent medical visits, diagnostic tests, and potential hospitalizations further adds to the complexity of care. These challenges highlight the need for multidisciplinary, patient-centered care models that integrate hepatology, endocrinology, and cardiovascular management.

## 5. Management of MASLD in the Context of Viral Hepatitis

Managing patients with dual MASLD and viral hepatitis is complex due to the interplay of metabolic and viral factors. Lifestyle modifications, including weight loss, dietary changes, and physical activity, are fundamental to MASLD management and help reduce fibrosis risk in viral hepatitis [11]. Structured lifestyle programs, such as the Diabetes Prevention Program, have shown efficacy in mitigating disease progression [37]. Additionally, interventions tailored to cultural and regional dietary practices may enhance patient adherence. Emerging agents targeting steatosis and inflammation, such as GLP-1 receptor agonists, offer promise [38]. Combination therapies targeting metabolic pathways are currently under investigation. A notable example is the randomized phase II trial by Alkhouri et al., which evaluated the safety and efficacy of combining semaglutide (a GLP-1 receptor agonist), cilofexor (a farnesoid X receptor agonist), and firsocostat (an acetyl-CoA carboxylase inhibitor) in patients with histologically confirmed steatohepatitis and stage F2–F3 fibrosis. The study demonstrated significant reductions in liver fat content and improvements in liver enzymes, particularly in the triple-therapy group. However, the study excluded patients with chronic viral hepatitis (HBV or HCV), and therefore did not assess effects on viral fibrogenic pathways [39].

The integration of multidisciplinary teams specializing in metabolic, viral, and liver-specific management is essential for optimizing clinical outcomes. Regular surveillance for HCC and the progression of fibrosis remains a cornerstone of comprehensive care. Digital health solutions like telemedicine and remote monitoring are reshaping traditional care models [40]. These innovations enhance access to specialist services, particularly in resource-constrained settings, and represent a critical advancement in the equitable delivery of healthcare.

## 6. Future Directions and Research Gaps

The intersection of MASLD and viral hepatitis presents numerous challenges and opportunities for further research and improvement in clinical management. One of the most pressing areas is the development of non-invasive biomarkers capable of accurately assessing fibrosis progression in patients with dual etiologies. While advanced methodologies such as extracellular vesicle profiling and metabolomics hold great promise for improving diagnostic precision, current tools like FibroScan remain limited. Despite being widely used to assess liver stiffness, FibroScan struggles to reliably differentiate between intermediate stages of fibrosis and is less accurate in patients with obesity or acute inflammation. In this context, machine learning and artificial intelligence represent an exciting frontier in hepatology. The ability to analyze large-scale patient datasets could enhance predictive models, enabling more personalized and effective disease management. By integrating traditional biomarkers with novel approaches, machine learning could refine the assessment of disease progression, offering a more comprehensive and nuanced understanding of fibrosis in complex hepatic conditions. Ultimately, advancing the field will require the development and validation of more precise biomarkers that can reliably guide clinical decision-making in patients with multifactorial liver diseases.

Beyond diagnostics, public health interventions are crucial in addressing the dual burden of MASLD and viral hepatitis. Implementing region-specific programs that seamlessly integrate viral screening with metabolic risk assessment is essential to improve early detection and timely intervention. However, significant challenges remain, including resource allocation, public awareness, and the need for stronger healthcare infrastructure. To overcome these barriers, public health initiatives must prioritize education and outreach efforts that emphasize the importance of early screening and preventive measures. Leveraging media platforms, social networks, and community centers can help disseminate information tailored to different linguistic and cultural contexts, ensuring broad accessibility. Additionally, expanding hepatitis B vaccination programs and integrating them with other health promotion activities could significantly increase uptake rates. Collaborations with schools, workplaces, and religious institutions can further enhance engagement, fostering a more proactive approach to disease prevention.

Lifestyle modifications remain a cornerstone in MASLD management and are equally critical in the context of viral hepatitis. However, the effectiveness of these interventions is closely tied to socioeconomic context and resource availability. In many low-income or underserved regions, access to healthy and affordable food options, safe environments for physical activity, and culturally tailored health education remains limited. This gap in accessibility makes it clear that lifestyle interventions alone may not be sufficient to address the growing global burden of MASLD. Therefore, public health strategies must prioritize equitable access to these resources by adapting to local infrastructure and addressing community-specific needs. For example, in some regions, partnerships with local businesses, fitness centers, or community leaders can help promote healthier behaviors and choices. However, such efforts must be underpinned by broader systemic changes that include providing subsidies for healthy food, urban planning that facilitates walkability and active transportation, and scalable digital health solutions that bridge the gap for underserved populations.

Moreover, a global perspective demands that interventions be flexible, context-sensitive, and rooted in health equity to ensure that solutions are applicable across different socioeconomic and cultural settings. Public health interventions should not only focus on behavioral change but also seek to reduce the barriers to healthier lifestyles, including food insecurity, inadequate physical activity spaces, and limited healthcare access. Global efforts must account for the diverse challenges faced by populations worldwide, from high-income urban centers to low-resource rural areas. Only with a holistic, inclusive approach can we truly mitigate the growing prevalence of MASLD and its associated complications, including its impact on viral hepatitis patients.

Looking ahead, the future of MASLD and viral hepatitis management depends on a comprehensive approach that integrates scientific advancements with innovative public health strategies. The convergence of emerging technologies, community-based interventions, and well-structured prevention policies holds the potential to transform the landscape of these diseases. However, for these efforts to be successful, sustained investment and coordinated collaboration among governments, healthcare institutions, and local communities will be essential. The road ahead is long, but with the right commitment and strategic planning, we can move toward a healthcare model that is more equitable, efficient, and responsive to the needs of patients. The challenge is immense, yet the opportunity to redefine the trajectory of MASLD and viral hepatitis is within reach.
